# Outcomes of patients with coronavirus disease versus other lung infections requiring venovenous extracorporeal membrane oxygenation

**DOI:** 10.1016/j.heliyon.2023.e17441

**Published:** 2023-06-17

**Authors:** Boris Kuzmin, Arevik Movsisyan, Florian Praetsch, Thomas Schilling, Anke Lux, Mohammad Fadel, Faranak Azizzadeh, Julia Crackau, Olaf Keyser, George Awad, Thomas Hachenberg, Jens Wippermann, Maximilian Scherner

**Affiliations:** a Department of Cardiothoracic Surgery, University Hospital, Magdeburg, Germany; b Department of Anesthesiology and Intensive Care Medicine, University Hospital, Magdeburg, Germany; c Institute of Biometry and Medical Informatics, University Hospital, Magdeburg, Germany

**Keywords:** Viral pneumonia, Acute respiratory failure, Extracorporeal membrane oxygenation, Bacterial superinfection, Cerebral bleeding

## Abstract

**Background:**

Patients with Coronavirus Disease (COVID-19) often develop severe acute respiratory distress syndrome (ARDS) requiring prolonged mechanical ventilation (MV), and venovenous extracorporeal membrane oxygenation (V–V ECMO).

Mortality in COVID-19 patients on V–V ECMO was exceptionally high; therefore, whether survival can be ameliorated should be investigated.

**Methods:**

We collected data from 85 patients with severe ARDS who required ECMO support at the University Hospital Magdeburg from 2014 to 2021. The patients were divided into the COVID-19 group (52 patients) and the non-COVID-19 group (33 patients). Demographic and pre-, intra-, and post-ECMO data were retrospectively recorded. The parameters of mechanical ventilation, laboratory data before using ECMO, and during ECMO were compared.

**Results:**

There was a significant difference between the two groups regarding survival: 38.5% of COVID-19 patients and 63.6% of non-COVID-19 patients survived 60 days (p = 0.024). COVID-19 patients required V–V ECMO after 6.5 days of MV, while non-COVID-19 patients required V–V ECMO after 2.0 days of MV (p = 0.048). The COVID-19 group had a greater proportion of patients with ischemic heart disease (21.2% vs 3%, p = 0.019). The rates of most complications were comparable in both groups, whereas the COVID-19 group showed a significantly higher rate of cerebral bleeding (23.1 vs 6.1%, p = 0.039) and lung bacterial superinfection (53.8% vs 9.1%, p = <0.001).

**Conclusion:**

The higher 60-days mortality among patients with COVID-19 with severe ARDS was attributable to superinfection, a higher risk of intracerebral bleeding, and the pre-existing ischemic heart disease.

## 1 Introduction

The coronavirus disease (COVID-19) pandemic has continued into its fourth year, with the next variant of the coronavirus [1]. Although most patients with COVID-19 have moderate symptoms and recover quickly, many develop severe respiratory failure (severe acute respiratory syndrome coronavirus 2 (SARS-CoV-2)) with acute respiratory distress syndrome (ARDS) and require long-term mechanical ventilation (MV) and a long stay in the intensive care unit (ICU) [2]. Mortality in patients with COVID-19 who develop severe ARDS and require MV is extremely high [3]. Patients with severe pneumonia in 10.5–15% needed extracorporeal membrane oxygenation (ECMO) [[4], [5], [6]]. This attaches great importance to the potential accessibility of venovenous ECMO (V–V ECMO) because it can be a life-saving organ support. ECMO can be indicated in patients with COVID-19, if ARDS is refractory to optimal conventional management, such as lung protective ventilation, prone position, neuromuscular blockade, and ventilation volume optimization [7]. Patients who received ECMO support fulfilled ARDS criteria and one of the following criteria for disease severity, despite optimized ventilation (fraction of inspired oxygen [FiO2] ≥80%, tidal volume set at 6 mL/kg predicted bodyweight, and positive end-expiratory pressure [PEEP] ≥10 cm of water): (1) partial pressure of arterial oxygen (pO2) over a FiO2 ratio of less than 50 mm Hg for more than 3 h; (2) pO2/FiO2 less than 80 mm Hg for more than 6 h; or (3) arterial blood pH less than 7.25 with a partial pressure of arterial carbon dioxide (pCO2) of 60 mm Hg or more for 6 h or more [8]. The reports on the effectiveness of ECMO in COVID-19 patients are controversial, and the mortality rate fluctuates within a wide range (37.4–73%) [[9], [10], [11]]. Organ support with ECMO and COVID-19 are often associated with synergistic changes in the hematological and inflammatory status of patients [12,13]. Possible risk factors (laboratory and clinical indicators and ventilation parameters) have not yet been sufficiently described in the literature. This study aimed to compare COVID-19 and non-COVID-19 ICU patients requiring ECMO to assess for possible COVID-19-specific mortality risk factors and complications.

## 2 Materials and methods

### 2.1 Study design

This retrospective cohort study used databases of ICU admissions from 2014 to 2021 in University Hospital Magdeburg. The Ethics Committee of the Otto-von-Guericke University in Magdeburg approved this observational retrospective study (Az 48/22).

Patients were selected if they received V–V ECMO by reason of ARDS as a result of pneumonia. Patients were excluded if they died less than 48 h after the initiation of ECMO, if they had severe trauma, acute pancreatitis, and recent cardiac surgery or lung surgery with partial lung resection.

Baseline covariates included demographics (age, sex), comorbidities, admission length before ECMO, mechanical ventilation before ECMO, and the sepsis-related organ failure assessment (SOFA) score. Time-varying covariates included ventilation and laboratory parameters before, and during ECMO support, as well as complications during ECMO support.

### 2.2 ECMO settings

Three different ECMO systems were used: CardioHelp, Rotaflow I, and Rotaflow II (Getinge AB, Rastatt, Germany). To connect the ECMO to the patient’s circulation, we used bi-femoral or femoro-jugular cannulation (in the right internal jugular vein). HLS or PLS cannulas (Getinge AB, Rastatt, Germany) were used. PTT was maintained using unfractionated heparin or argatroban for 40–60 s. The ECMO parameters varied in patients: blood flow 4.0–6.4 L/min, gas flow 3–8 L/min, rotational speed 3000–4500 rotations/min. The fraction of inspired oxygen (FiO2) on ECMO device was initially always 80–100% and reduced subsequently as the patient’s condition improves.

### 2.3 Outcomes

The primary outcome was a 60-day survival rate after ECMO application. Secondary outcomes included length of stay before ECMO, complications, major bleeding events (cerebral, gastrointestinal, hemothorax, cannulation-side bleeding, endobronchial, retroperitoneal, neck-bleeding [tracheostoma], chest-wall hematoma), procedures such as circuit change, ECMO duration, ventilator recordings, and laboratory parameters throughout V–V ECMO. These values and incidences were compared in an analysis of non-COVID-19 and COVID-19 groups.

There were no predefined criteria in our clinic for the termination of ECMO. This decision was made for each case by an interdisciplinary treatment team. In some cases, the ECMO support was terminated by intensive care physicians, pulmonologists, and cardio-thoracic surgeons because of severe complications (e.g., cerebral hemorrhage, pulmonary hemorrhage, or massive hemothorax, which were not treatable. In others, long-term ECMO support was not continued for more than 3–4 weeks for severe COVID-19, because chest CT showed that the lungs had not recovered, and the organ structure was destroyed. ECMO support was then terminated, in particular, only upon the development of another new complication.

### 2.4 Statistical analysis

Baseline patient demographics (age, sex, body mass index, comorbidities), ventilator recordings, ECMO duration, laboratory parameters, as well as complications (bleeding events, stroke, hospital-acquired infections, acute kidney injury, acute liver failure, pneumothorax, pulmonary embolism, abdominal compartment syndrome, explorative laparotomy/ischemic gut, thrombocytopenia, lung abscess, pleural empyema, tracheal rupture, pneumomediastinum) and outcomes were compared between the two groups using SPSS version 28 (IBM, Armonk, New York, USA). Graphs were created using Microsoft® Excel® and add‐in XLSTAT for Windows 10. Normality was examined using Kholmogorov-Smirnov tests. The two-tailed independent *t*-test or Mann-Whitney *U* test was performed for univariate comparison of numerical variables. Categorical variables were analyzed using the chi-squared or Fisher’s exact test. Data are presented as mean (± standard deviation) for numerical variables with a normal distribution or median (interquartile range; IQR) for numerical variables without a normal distribution. Categorical data are presented as number (percentage). The significance level was set at α = 0.05. To estimate the survival curve, we used the Kaplan-Meier method. Multivariate logistic regression was used to identify the independent predictors of 60-days mortality of the entire population. We processed variables before V–V ECMO and variables such as complications during V–V ECMO by using the logistic regression method [14]. The variables showing a p ≤ 0.1 in the logistic regression process were re-tested by the logistic regression method with an inclusion selection procedure to build the final prediction model.

## 3 Results

### 3.1 Patients

**Fig. 1:**
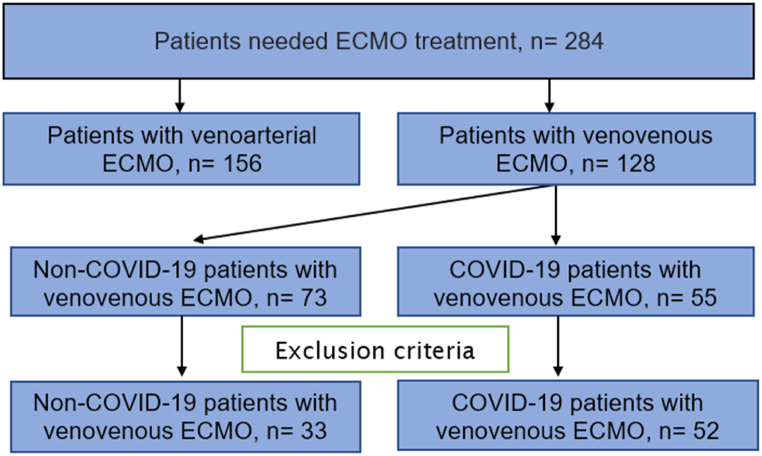
Study design.

From 2014 to 2021 284 patients needed ECMO support in the University Hospital Magdeburg. Of these 156 required venoarterial ECMO and 128 V–V ECMO. Among the 128 patients with V–V ECMO, 55 had COVID-19, and 73 needed V–V ECMO because of non-COVID-19 ARDS. After applying the exclusion criteria, a total of 85 critically ill patients with ARDS on V–V ECMO were included in this study. Of these, 52 patients had COVID-19, and 33 had other infectious lung disease (Fig. 1).

The mean age was 57.0 ± 13.4 years (52.8 ± 15.7 years in non-COVID-19 group; 59.7 ± 11.0 years in COVID-19 group, p = 0.212). Most patients were men (64/85; 75.3%), which was the same ratio for both groups. The COVID-19 group was characterized by higher weight (Table 1). Furthermore, COVID-19 patients required V–V ECMO after 6.5 (2.0–11.0) days of invasive pressure controlled MV, while the non-COVID-19 patients needed V–V ECMO within 2.0 (1.0–6.0) days of MV (p = 0.048). The number of hospital days before V–V ECMO was also considerably higher in the COVID-19 group. There was no correlation between mortality and the place of the initial admission for entire population. Of the 40 patients initially admitted to the University Hospital Magdeburg, 19 patients survived ARDS with ECMO support, whereas of 45 patients transferred to the University hospital Magdeburg, 22 patients survived V–V ECMO (mortality rate 52.5% vs. 51.1%, p = 0.898).

**Table 1:** Baseline characteristics for the entire population and the COVID-19 and non-COVID-19 groups.

Baseline characteristics	Overall, n = 85	non-Covid-19 group, n = 33	Covid-19 group, n = 52	p-value
Age	57.0 ± 13.4	52.8 ± 15.7	59.7 ± 11.0	0.212
Male	64 (75.3%)	25 (75.8%)	39 (75%)	0.937
BMI	29.3 (24.6–33.8)	27.6 (23.3–31.3)	29.4 (27.4–34.6)	**0.033**
Hospital day before ECMO	10.0 (4.0–14.0)	5.3 (2.2–13.0)	10.6 (7.0–14.7)	**0.013**
Mechanical ventilation before ECMO (day)	4.0 (1.0–9.0)	2.0 (1.0–6.0)	6.5 (2.0–11.0)	**0.048**
Chronic kidney disease	11 (12.9%)	4 (12.1%)	7 (13.5%)	0.857
Autoimmune disease	4 (4.7%)	3 (9.1%)	1 (1.9%)	0.128
Hematological malignancy	7 (8.2%)	5 (15.2%)	2 (3.8%)	0.064
Hypertension	52 (61.2%)	16 (48.5%)	36 (69.2%)	0.056
Ischemic heart disease	12 (14.1%)	1 (3%)	11 (21.2%)	**0.019**
Diabetis	25 (29.4%)	7 (21.2%)	18 (34.6%)	0.186
Chronic lung disease	14 (16.5%)	5 (15.2%)	9 (17.3%)	0.793
Immuncompromised status	13 (15.3%)	11 (33.3%)	2 (3.8%)	**<0.001**
Acute liver failure before ECMO	6 (7.1%)	5 (15.2%)	1 (1.9%)	**0.020**
SOFA score	9.3 ± 3.4	9.7 ± 3.7	9.1 ± 3.2	0.411

**Fig. 2:**
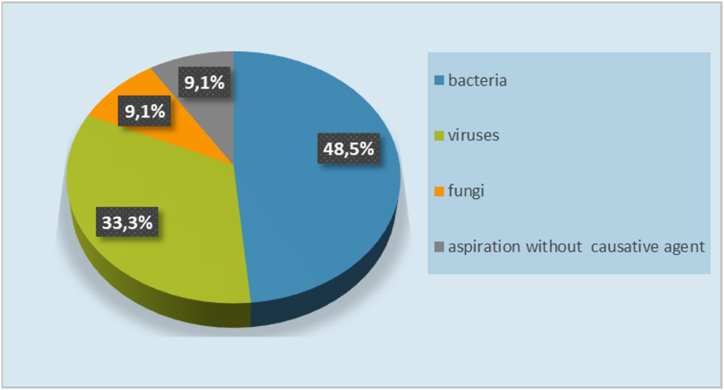
Causative agents in the non-COVID-19 group (%).

There were no significant differences in the prevalence of hypertension, chronic kidney disease, autoimmune disease, hematological malignancy, chronic lung disease before admission, or diabetes mellitus between the two groups. There were neither difference in SOFA score. The following distinguishing characteristics were the differences in the associated diseases such as ischemic heart disease, which was more often found in COVID-19 patients whereas immunocompromised status and acute liver failure before ECMO emerged more often in the non-COVID-19 group. We accepted that these differences were due to various disease characteristics in the non-COVID-19 group. Namely, 16 patients (48.5%) in this group initially suffered ARDS due to severe bacterial pneumonia; seven (21.2%) of them after aspiration. The following bacteria were identified in this group: *Klebsiella pneumoniae/oxytoca* (3, 9.1%), *Pseudomonas aeruginosa* (4, 12.1%), *Proteus mirabilis* (3, 9.1%), *Streptococcus pneumoniae* (3, 9.1%), *Escherichia coli* (3, 9.1%), *Acinetobacter baumannii* (2, 6.1%), *Enterobacter cloacae* (1, 3.0%). Eleven (33.3%) patients were diagnosed with viral pneumonia secondary to influenza A virus (2, 6.1%), influenza B virus (1, 3.0%), respiratory syncytial virus (1, 3.0%), cytomegalovirus (4, 12.1%), or Herpes Simplex virus (5, 15.2%). In three cases, Herpes simplex virus and Cytomegalovirus were found as a second causative agent. Three (9.1%) patients developed fungal pneumonia caused by *Pneumocystis jirovecii* and *Candida*, and in three patients, there was aspiration with no causative agent. The presence of low pathogenic viruses and fungi in the non-COVID-19 group is explained by the fact that 33.3% of these patients were immunocompromised. The causative agents in the non-COVID-19 group are shown in Fig. 2.

We noted differences in the laboratory parameters between the groups. In particular, the non-COVID-19 group was characterized by more severe hypoxemia, while the COVID-19 group suffered from greater hypercapnia, albite, according to statistical tests, this was not significant (Table 2). Higher procalcitonin level in the non-COVID-19 group correlate with a higher incidence of sepsis in the case of bacterial infection, because in patients with COVID-19 pneumonia a rise in procalcitonin was observed only in bacterial superinfection [15,16]. We also explain the differences in hemostasis (platelet count, INR, fibrinogen [g/L]) by the presence of more liver disorders in the non-COVID-19 group [17,18].

**Table 2:** Laboratory parameters before V–V ECMO application of the entire population and for the COVID-19 and non-COVID-19 groups.

Laboratory parameters before venovenous ECMO				
	Overall, n = 85	non-Covid-19 group, n = 33	Covid-19 group, n = 52	p-value
pO2 (mmHg)	68.0 (58.0–79.5)	62.0 (53.5–70.0)	71.5 (61.0–90.0)	**0.001**
pCO2 (mmHg)	70.0 (53.0–90.0)	61.0 (50.5–82.3)	77.5 (56.4–99.0)	0.125
Hemoglobin (mmol/l)	6.6 ± 1.2	6.5 ± 1.2	6.8 ± 1.3	0.216
Leucocyte (10^9^/l)	15.4 (9.4–20.9)	17.1 (10.4–24.6)	15.1 (9.3–20.2)	0.446
Platelet count (10^9^/l)	253.3 ± 133.5	193.6 ± 151.6	294.6 ± 103.8	**0.002**
CRP (mg/L)	274.9 ± 127.1	253.6 ± 139.1	244.2 ± 120.2	0.752
Procalcitonin (ng/ml)	1.1 (0.5–3.0)	2.8 (1.3–15.9)	0.7 (0.3–1.4)	**<0.001**
PTT (s)	32.0 (29.9–40.7)	33.0 (30.0–39.5)	31.6 (29.0–40.7)	0.419
INR	1.20 (1.13–1.29)	1.23 (1.17–1.46)	1.17 (1.11–1.24)	**0.010**
D-Dimer (mg/l)	6.9 ± 6.2	8.3 ± 6.5	6.1 ± 6.0	0.130
Fibrinogen (g/l)	6.1 ± 2.2	5.2 ± 2.3	6.7 ± 2.0	**0.004**
ALT (μmol/ls)	0.6 (0.4–1.7)	0.6 (0.4–2.1)	0.6 (0.4–1.6)	0.828
AST (μmol/ls)	1.1 (0.7–1.7)	1.2 (0.8–1.8)	0.9 (0.7–1.6)	0.425
Creatinine (μmol/l)	99.0 (66.5–139.0)	99.5 (71.0–180.0)	96.5 (62.8–131.3)	0.230
Total bilirubin (μmol/l)	12.2 (7.8–26.1)	13.5 (8.2–36.2)	10.8 (7.6–19.8)	0.123

As regards ventilator parameters (Table 3), we observed higher respiratory pressures before V–V ECMO application in the non-COVID-19 group (Peak inspiratory pressure [PIP)] [cmH2O], Mean airway pressure [cmH2O]), while a significant drop in respiratory pressures in this group of patients in 24 h after V–V ECMO application (PIP [cmH2O], Mean airway pressure [cmH2O], Positive end-expiratory pressure [PEEP] [cmH2O]). Thus, to ensure a minimal tidal volume (ml/kg ideal body weight [IBW]) in order to prevent total lung collapse, the respiratory pressures (primarily PEEP) in COVID-19 patients had to remain high.

**Table 3:** Ventilator parameters before ECMO and after 24 h of ECMO support of the entire population and for the COVID-19 and non-COVID-19 groups.

Ventilator parameters before venovenous extracorporeal membrane oxygenation				
	Overall, n = 85	non-Covid-19 group, n = 33	Covid-19 group, n = 52	p-value
PaO_2_/FiO_2_ (mmHg)	72.0 (58.2–113.0)	62.2 (53.0–78.0)	93.5 (67.0–131.0)	**0.001**
Peak inspiratory pressure (cmH_2_O)	34.0 (31.0–37.0)	36.0 (33.0–40.0)	33.5 (30.0–36.0)	**0.011**
Mean airway pressure (cmH_2_O)	23.2 ± 3.6	24.4 ± 4.5	22.4 ± 2.7	**0.025**
PEEP (cmH_2_O)	15.0 (12.0–16.0)	13.0 (11.0–16.0)	15.0 (13.0–16.0)	0.148
Measured tidal volume (ml/kg IBW)	5.0 (3.9–6.7)	5.8 (4.0–7.3)	5.0 (3.9–5.6)	0.092
Dynamic compliance (ml/cmH_2_O)	24.0 ± 8.8	21.8 ± 9.3	25.4 ± 8.2	0.071
**Ventilator parameters after 24 h of venovenous extracorporeal membrane oxygenation**				
Peak inspiratory pressure (cmH_2_O)	27.0 (25.0–30.0)	26.0 (24.8–27.0)	29.0 (26.0–31.0)	**0.001**
Mean airway pressure (cmH_2_O)	20.0 (17.0–21.0)	17.4 (16.9–20.0)	20.0 (18.4–22.0)	**0.002**
PEEP (cmH_2_O)	14.0 (12.0–15.0)	12.0 (9.9–15.0)	15.0 (13.8–15.0)	**0.006**
Measured tidal volume (ml/kg IBW)	3.2 ± 1.5	3.0 ± 1.30	3.3 ± 1.5	0.292
Dynamic compliance (ml/cmH_2_O)	20.9 ± 10.2	18.4 ± 7.9	22.4 ± 11.1	0.058

### 3.2 Primary and secondary outcomes

**Fig. 3:**
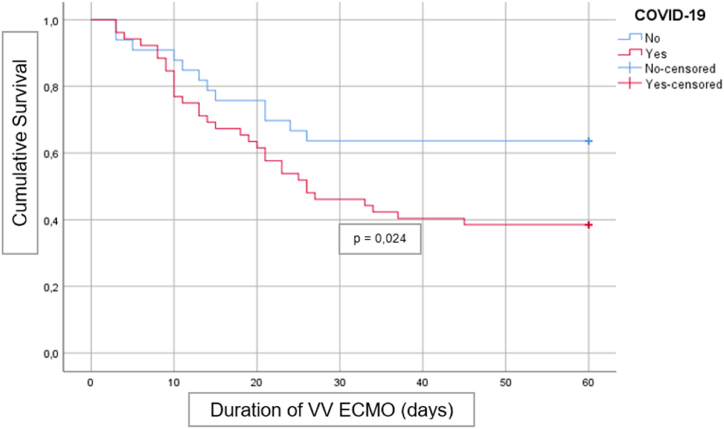
Kaplan–Meier curve between the COVID-19 (n = 52) and non-COVID-19 (n = 33) groups during the first 60 days after ECMO-initiation. ECMO, extracorporeal membrane oxygenation.

Overall 60-days mortality for the entire cohort was 48.2% (n = 41) (Table 4). There was a significant difference between the two groups in survival: 38.5% of COVID-19 patients and 63.6% of non-COVID-19 patients survived 60 days (p = 0.024) (Fig. 3).

**Table 4:** Survival, complications, and characteristics of the therapy during V–V ECMO of the entire population and for the COVID-19 and non-COVID-19 groups.

Characteristics	Overall, N = 85	Non-COVID-19 group, n = 33	COVID-19 group, n = 52	p-Value
Survivors	41 (48.2%)	21 (63.6%)	20 (38.5%)	0.024
Site of cannulation:				
- Femoro-jugularis	17 (20%)	8 (24.2%)	9 (17.3%)	0.436
- Femoro-femoralis	68 (80%)	25 (75.8%)	43 (82.7%)	0.436
Circuit change of ECMO	15 (17.6%)	4 (12.1%)	11 (21.2%)	0.287
Major bleeding events	47 (55.3%)	17 (51.5%)	30 (57.7%)	0.577
- cerebral	14 (16.5%)	2 (6.1%)	12 (23.1%)	0.039
- gastrointestinal	11 (12.9%)	4 (12.1%)	7 (13.5%)	0.857
- hemothorax	9 (10.6%)	3 (9.1%)	6 (11.5%)	0.721
- cannulation side	11 (12.9%)	6 (18.2%)	5 (9.6%)	0.251
- endobronchial	4 (4.7%)	0	4 (7.7%)	0.103
- retroperitoneal	2 (2.4%)	0	2 (3.8%)	0.254
- neck-bleeding (tracheostoma)	5 (5.9%)	3 (9.1%)	2 (3.8%)	0.317
- chest-wall hematome	3 (3.5%)	1 (3%)	2 (3.8%)	0.843
Stroke	2 (2.4%)	0	2 (3.8%)	0.254
Acute kidney injury requiring dialysis during ECMO	30 (35.3%)	14 (42.4%)	16 (30.8%)	0.273
Acute kidney injury without dialysis	12 (14.1%)	2 (6.1%)	10 (19.2%)	0.089
Acute liver failure	11 (12.9%)	4 (12.1%)	7 (17.1%)	0.857
Lung bacterial superinfection	31 (36.5%)	3 (9.1%)	28 (53.8%)	<0.001
Abdominal compartment syndrome	9 (10.6%)	4 (12.1%)	5 (12.2%)	0.714
Pneumothorax	8 (9.4%)	4 (12.1%)	4 (9.8%)	0.495
Lung abscess or pleural empyema	7 (8.2%)	4 (12.1%)	3 (7.3%)	0.299
Tracheal rupture, pneumomediastinum	3 (3.5%)	1 (3.0%)	2 (4.9%)	0.843
Explorative laparotomy/ischemic gut	7 (8.2%)	5 (15.2%)	2 (4.9%)	0.065
Lung emboly	4 (4.7%)	2 (6.1%)	2 (4.9%)	0.638

Duration of the V–V ECMO in all patients was to some extent longer in the COVID-19 group (13.0 [8.0–17.0] vs. 16.5 [10.0–24.0], p = 0.099), albeit the difference in the duration of the ECMO support among survivors was statistically significant in this group (13.0 [8.0–16.0] vs. 17.0 [12.0–24.0], p = 0.035).

There were no differences in the site of cannulation or circuit change during V–V ECMO (Table 4). The rates of complications such as stroke, bacteremia, renal failure requiring dialysis, abdominal compartment syndrome, pneumothorax, lung abscess or pleural empyema, tracheal rupture/pneumomediastinum, and pulmonary embolism were comparable in both groups, whereas we detected significant differences in cerebral bleeding (6.1% vs. 23.1%, p = 0.039) and lung bacterial superinfection (9.1% vs. 53.8%, p = <0.001). We define plenty of hemorrhagic events as major, but life-threatening bleeding prevailed in the COVID-19 group (cerebral bleeding, as described above; endobronchial bleeding, 0% vs. 7.7% or retroperitoneal bleeding, 0% vs. 3.8%). COVID-19 patients suffered more from acute kidney injury without dialysis (6.1% vs. 19.2%, p = 0.089). These patients were in stage 1–2 acute renal failure according to Acute Kidney Injury Network classification ([1] increase in serum creatinine of more than or equal to 26.4 μmol/l or increase to more than or equal to 150%–200% [1.5- to 2-fold] from baseline; or [2] increase in serum creatinine to more than 200%–300% [> 2- to 3-fold] from baseline), but they were still passing sufficient urine [19]. Non-COVID-19 patients more often underwent exploratory laparotomies because of an acute abdomen (15.2% vs. 4.9%, p = 0.065).

**Fig. 4:**
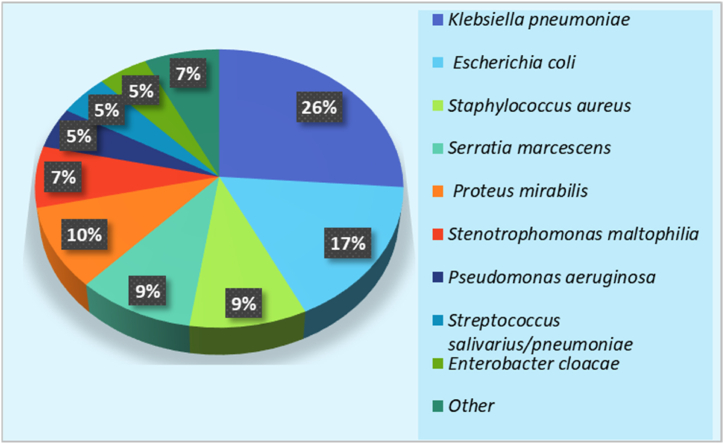
Bacterial spectrum in COVID-19 patients with superinfection (% in the patients with superinfection).

Among the patients with bacterial superinfection (53.8%) in the COVID-19 group, only one bacterial species was cultured from the lungs in 18 (34.6%) patients, two in six (9.1%) patients, and three bacteria in four (7.7%) patients. On average, pulmonary pathogens were detected on day 6 after intubation and reflect the spectrum of hospital-acquired pneumonia/ventilator-associated pneumonia. The bacterial spectrum that caused superinfection in the COVID-19 patients are presented in Fig. 4.

The laboratory parameters seven days after ECMO application were similar, except for a lower d-dimer level, longer PTT, and to some extent higher CRP in the COVID-19 group (Table 5). All respiratory pressures seven days after ECMO application were significantly higher in the COVID-19 group, whereas measured tidal volume, and dynamic compliance were crucially lower (Table 6).

**Table 5:** Laboratory parameters and ventilator parameters during ECMO support of the entire population and for the COVID-19 and non-COVID-19 groups.

Laboratory parameters after seven days of the V–V ECMO support				
	Overall, n = 73	Non-COVID-19 group, n = 26	COVID-19 group, n = 47	p-value
pO2 (mmHg)	74.4 ± 18.0	78.6 ± 21.1	72.2 ± 13.7	0.181
pCO2 (mmHg)	44.4 ± 6.8	42.8 ± 6.1	45.3 ± 7.1	0.121
Hemoglobin (mmol/l)	6.2 ± 0.6	6.2 ± 0.7	6.2 ± 0.6	0.589
Leucocyte (10^9^/l)	9.3 (6.7–14.6)	12.4 (8.2–19.5)	8.8 (6.3–12.5)	0.060
Platelet count (10^9^/l)	103.0 (84.0–155.5)	91.0 (81.5–150.8)	108.0 (87.5–156.0)	0.201
CRP (mg/L)	157.9 ± 102.6	130.3 ± 86.8	173.2 ± 108.3	0.070
Procalcitonine (ng/ml)	0.7 (0.4–1.8)	0.9 (0.4–2.1)	0.6 (0.4–1.5)	0.399
PTT (s)	56.9 ± 9.0	54.3 ± 7.5	58.3 ± 9.6	0.053
INR	1.20 (1.12–1.35)	1.21 (1.11–1.43)	1.20 (1.12–1.33)	0.931
ATIII (%)	80.4 ± 15.4	78.2 ± 14.3	81.6 ± 16.0	0.366
D-Dimer (mg/l)	9.6 (4.8–17.7)	17.4 (9.1–20.0)	7.3 (3.4–13.2)	**0.002**
Fibrinogen (g/l)	4.7 (3.9–6.9)	4.5 (3.8–6.1)	5.3 (4.0–7.0)	0.238
ALT (μmol/ls)	0.7 (0.4–1.2)	0.7 (0.4–1.3)	0.6 (0.4–1.1)	0.686
AST (μmol/ls)	1.0 (0.6–1.6)	0.9 (0.6–1.7)	1.0 (0.6–1.5)	0.482
Creatinine (μmol/l)	63.0 (45.5–88.5)	61.0 (45.0–73.5)	64.0 (46.5–102.0)	0.410
Total bilirubin (μmol/l)	25.4 (11.3–73.7)	20.4 (11.8–60.8)	31.0 (10.5–73.7)	0.913

**Table 6:** Ventilator parameters during ECMO support of the entire population and for the COVID-19 and non-COVID-19 groups.

Ventilator parameters after seven days of the V–V ECMO support				
	Overall, n = 73	Non-COVID-19 group, n = 26	COVID-19 group, n = 47	p-value
Peak inspiratory pressure (cmH_2_O)	28.4 ± 4.7	25.1 ± 5.4	30.1 ± 3.2	**<0.001**
Mean airway pressure (cmH_2_O)	19.6 ± 3.7	17.1 ± 4.4	20.9 ± 2.5	**<0.001**
PEEP (cmH_2_O)	14.0 (12.0–15.8)	10.0 (8.0–14.0)	15.0 (13.0–15.5)	**0.009**
Measured tidal volume (ml/kg IBW)	3.4 (2.1–5.0)	4.4 (3.0–5.0)	3.1 (2.0–4.3)	**0.028**
Dynamic compliance (ml/cmH_2_O)	21.9 ± 11.2	26.1 ± 11.3	19.7 ± 10.6	**0.022**

### 3.3 Prediction model of hospital death

We performed logistic regression and identified the relevant prediction model. According to the results of bivariate analysis, the COVID-19 (OR: 2.800; 95% confidence interval [CI]: 1.135–6.907, p = 0.025), the bacterial superinfection (OR: 3.556; 95% CI: 1.380–9.163; p = 0.009), high CRP level (mg/L) on the 7th day of V–V ECMO (OR: 0.991; 95% CI: 0.986–0.996, p = 0.001), low dynamic compliance (ml/cmH2O) after seven days of V–V ECMO (OR: 1.047; 95% CI: 1.002–1.094, p = 0.050), and the need for high mean airway pressure during ECMO (cmH2O) (OR: 0.857; 95% CI: 0.747–0.983, p = 0.028) were identified as independent predictors of 60-days mortality in adult patients who received V–V ECMO because of severe ARDS. Among numerous other parameters the presence of acute kidney injury without dialysis (OR: 3.257; 95% CI: 0.815–13.011, p = 0.095), the existence of ischemic heart disease (OR: 3.257; 95% CI: 0.815–13.011; p = 0.095), and PTT (OR: 0.956; 95% CI: 0.906–1.009; p = 0.101) trended to have a significant effect, whereas the cerebral bleeding increased mortality with lower significance (OR: 1.851; 95% CI: 0.564–6.074, p = 0.310).

Subsequently, a multivariate analysis was performed with all variables that were significant or tended to be significant (p ≤ 0.1). According to the results of multivariate analysis, the bacterial superinfection (OR: 5.435; 95% CI: 1.148–25.729; p = 0.033), high CRP level on the seventh day of V–V ECMO (OR: 0.989; 95% CI: 0.982–0.997, p = 0,004), and ischemic heart disease (OR: 8.017; 95% CI: 1.002–64.443, p = 0.050) were significant. Other parameters weren’t strong significant: low dynamic compliance (ml/cmH2O) after seven days of V–V ECMO increased risk of death (OR: 1.062; 95% CI: 0.994–1.135, p = 0.077); acute kidney injury without dialysis reduced chances of survival by 4.7 times (OR: 4.658; 95% CI: 0.784–27.657, p = 0.091). The other parameters revealed no significant effect on survival in this regression analysis: PTT OR: 0.974; 95% confident interval (CI): 0.898–1.035, p = 0.314; the need for high airway pressure on the 7th day of V–V ECMO: OR: 1.038; 95% CI: 0.848–1.270, p = 0.717; the presence of COVID-19: OR: 0,390; 95% CI: 0,064–2,365, p = 0,306. The mortality prediction model explained 43.2% (Nagelkerke R2) of the variance in-hospital mortality and correctly classified 72.2% of the cases (sensitivity: 75.7%; specificity, 68.6%). The Hosmer-Lemeshow-Test assessed goodness-of-fit, indicating a good model fit (χ ^2^ (8) = 9.991, p = 0.266).

## 4 Discussion

In this retrospective single-center study of critically ill patients supported by V–V ECMO with infectious lung disease, we compared the outcomes of COVID-19 and non-COVID-19 infectious diseases.

Our study revealed that a 60-days survival in the non-COVID-19 group was significantly higher. Only a few reports have compared patients with COVID-19 on ECMO to patients with other infectious diseases on ECMO. Dave et al. also reported a higher mortality in COVID-19 (49% vs. 24%, p = 0.017), when compared to other viral infections [20]. Most of studies have compared COVID-19 patients on V–V ECMO to each other during different pandemic waves. The largest number of patients were in the study by Barbaro et al.; in their 4812 patients with COVID-19 on ECMO, the cumulative incidence of in-hospital mortality 90 days after ECMO initiation was 36.9%–58.9%, depending on the wave of the pandemic [21]. In studies from Germany and France, the overall 90-day mortality was 52–68%, again depending on the wave of the pandemic [22,23]. Barbaro and Dave demonstrated better survival in patients with COVID-19 compared with reports from France and Germany (including our results), apparently due to better patient selection; the average age in the first reports was 47–50 years compared to 59–61 in other reports [[20], [21], [22], [23]]. In addition, the group studied by Dave et al. did not include patients with ischemic heart disease; Barbaro et al. reported that only 3–5% of patients had a pre-existing heart disease in the COVID-19 group [20,21], whereas 21.1% of patients with COVID-19 in our study had pre-existing ischemic heart disease. In all these studies including ours, 73–83% of patients were male; male sex was an important driver of mortality risk in all patients with COVID-19, due to hormonal, inflammatory, immunological, and phenotypic differences, and severe disease patterns [24]. For patients with high-risk factors (e.g., older age and comorbidities, which are assessed individually) and when ECMO serves as “salvage ECMO,” determining the indication is difficult [25].

Even before initiation of V–V ECMO, patients in the two groups differed in the clinical course of the disease, especially regarding the time frame. Therefore, the illness burden of the patients with COVID-19 increased gradually, and they required V–V ECMO much later than the patients in the non-COVID-19 group. Nonetheless, patients with COVID-19 spent significantly longer periods of time on MV before ECMO cannulation (6.5 (2.0–11.0) days) with lower but still high respiratory pressures. Dave et al. also reported the difference in the length of MV in patients with COVID-19 vs patients with other viral pneumonia before ECMO, which were shorter as in our study (1.0 (0.25–3.0) vs 3.0 (1.5–4.5) days) [20]. Barbaro et al. reported MV before ECMO in COVID-19 patients in 4.3 (2.0–6.5) days, whereas in the study by Widmeier et al. in Germany, it was 7.8 (4–12.7) days. We can conclude that in Germany, contraindications to ECMO were excluded more liberally whereas we observe many patients who were on MV before ECMO for longer than 7 days, which was identified as a relative contraindication for cannulation for V–V ECMO by the Extracorporeal Life Support Organization [7]. Extended MV before V–V ECMO could be the most important factor for high mortality [7]. Wu et al. demonstrated an independent association between extended MV and in-hospital mortality in non-COVID-19 patients receiving V–V-ECMO for severe ARDS [26]. The duration of MV before ECMO cannulation did not significantly affect survival in our study, but this should still be considered in a study with more patients.

The ventilator parameters before ECMO initiation in COVID-19 patients were similar compared to the other studies [21,23]. Hence, we can conclude that the transition strategy from aggressive MV to ECMO was similar across these studies, as the maximum ventilation pressures (i.e., 33.0–37.0 cmH_2_O for PIP and 14.0–16.0 cmH_2_O for PEEP) no longer enabled appropriate blood gases.

In our study, we observed that respiratory pressures could be reduced in 24 h to a greater extent in non-COVID-19 patients (Table 3). This allows us to conclude that the applying ECMO in patients with COVID-19 enables small reduction in the ventilation pressures to ensure a minimal tidal volume and dynamic compliance to prevent total lung collapse. The minimal tidal volume and dynamic compliance were similar in both groups within 24 h of initiating ECMO despite marked differences in the respiratory pressures. A measured tidal volume and dynamic compliance have merely worsened in COVID-19 patients in 7 days of ECMO support despite how respiratory pressures remained significantly higher; in the non-COVID-19 group, these respiratory parameters improved considerably. We can infer that the lungs of patient with COVID-19 remained rigid due to inflammation and infiltration even after 7 days of ECMO support. Data on the effects of ventilation parameters in patients with COVID-19 on ECMO have not been presented in any study and are certainly of great interest, especially in relation to predicting survival. Studies pre-COVID-19 pandemic showed that the PIP of 42 cmH2O before the ECMO application predicted hospital mortality in adult patients [27]. Researchers assumed before the COVID-19 pandemic that driving pressure (the difference between plateau pressure and PEEP) is also a valuable indicator for assessing mortality [28,29]. Although driving pressure is a better airway pressure than peak inspiratory pressure to measure the pressure on the lungs during MV, we could not calculate this parameter because the data about ventilation parameters were incomplete in this retrospective study.

As a consequence of extended MV, the second most important factor can be bacterial superinfection. On average, we detected superinfection on day 6 after intubation, reflecting the spectrum of hospital-acquired pneumonia/ventilator-associated pneumonia resulting from prolonged MV, as has been shown in other studies [30]. The deterioration of illness and the requirement for V–V ECMO in COVID-19 patients can be allegedly caused by bacterial infections. The presence of superinfection was associated with longer ventilation times and increased ICU and total hospital LOS [30]. The rate of bacterial superinfection (53.8%) in the COVID-19 group was a supposedly crucial factor for increased mortality [13,31].

The obesity, hypertension and ischemic heart disease were the comorbidities which had the prevalence in the COVID-19 group also in our study. These disorders have a strong relationship with SARS-CoV-2 [32]. ACE2 enzyme was originally identified in 2003 as a receptor for SARS-CoV, so that the coronavirus enters the cells via this enzyme [33,34]. ACE2 inhibitors as well as angiotensin receptor blockers may stimulate the expression of the ACE2 receptor, which can increase susceptibility to SARS-CoV-2 infection [35]. The association between ischemic heart disease and COVID-19 is not precise, but this connection is presumed by the escalation of myocardial injury or due to ACE2 receptors’ presence on cardiac muscle cells, suggesting the potential involvement of the cardiovascular system in SARS-CoV-2 infection [32].

Another relevant finding of our study was the significantly more cerebral bleedings observed in the COVID-19 group, as outlined in other studies with a frequency of 5.9%–10.5% and the 92% mortality after this neurological event [36,37]. This distinction was observed even though the patients in the non-COVID-19 group had a more hypocoagulative status when V–V ECMO support was commenced. The coagulation spectrum after seven days of V–V ECMO support was roughly the same, except for significantly lower D-dimer levels and a more elongated PTT in COVID-19 patients. The role of the D-dimer remains controversial. The hypercoagulable state suggested by higher D-dimer levels in patients with COVID-19 can result in over-anticoagulation and devastating bleeding [38]. D-dimer levels may be partially elevated owing to the ECMO circuit [38]. There were no differences in thrombotic events between the two groups. As mentioned previously, PTT was supported by unfractionated heparin between 40 and 60 s, which has been described as safe [39]. However, we observed a tendency toward the upper limit in the COVID-19 group (58.3 ± 9.6 s), which might cause increased bleeding events. Forty percent of professionals from 98 centers in 30 different countries confirmed using higher doses of anticoagulants than usual in patients on ECMO during COVID-19 pandemic [40]. Future studies should examine whether these factors can cause additional bleeding events. A small study showed that a low PTT (45–55 s) significantly prevents major bleeding events [41].

The incidence of severe AKI requiring continuous renal replacement therapy (CRRT) while on V–V ECMO was described in 35–44% of patients [42,43]. In our study, 50% of COVID-19 patients suffered from acute renal failure: 19.2% weren’t on CRRT, and 30.2% required a dialysis. We found that acute renal failure, which was not treated with CRRT, was associated with increased patient mortality, and mostly affected COVID-19 patients. AKI is described as an independent in-hospital mortality risk factor in COVID-19 patients [44]. We attribute this phenomenon to the fact that only AKI without CRRT was shown in our study as an increased mortality factor due to the fact that all AKI contributes to high mortality, and we should not underestimate a timely commencement of dialysis. It is possible that the indications for CRRT were downgraded at the time of the COVID-19 pandemic because of the high strain on the healthcare system.

This study has several limitations, including the retrospective nature of the review and its single-center design, which may account for data collection errors, and moderate sample size. The clinical value is the strengths of our study. Upon comparison of patients with ARDS due to COVID-19 versus other infectious diseases, we saw the differences in the management of V–V ECMO for patients with COVID-19 (much longer ECMO support, problems related to anti-coagulation therapy, bacterial lung superinfection).

## 5 Conclusion

A mortality rate was considerably higher in the COVID‐19 population on ECMO compared with non-COVID‐19 patients. Bacterial superinfection and the presence of ischemic heart disease were the main predictors of increased mortality in this group of patients. The additional high mortality in the COVID‐19 group was associated with a higher risk of intracerebral bleeding.

## Author contribution statement

Boris Kuzmin: Conceived and designed the experiments; Analyzed and interpreted the data; Contributed reagents, materials, analysis tools or data; Wrote the paper.

Arevik Movsisyan; Faranak Azizzadeh; Julia Crackau; Olaf Keyser; George Awad: Contributed reagents, materials, analysis tools or data.

Florian Praetsch; Mohammad Fadel; Thomas Hachenberg; Jens Wippermann: Conceived and designed the experiments.

Thomas Schilling; Anke Lux: Analyzed and interpreted the data.

Maximilian Scherner: Conceived and designed the experiments; Analyzed and interpreted the data; Wrote the paper.

## Funding statement

The author(s) received no financial support for the research, authorship, and/or publication of this article.

## Data availability statement

Data will be made available on request.

## Declaration of competing interest

The authors declare that they have no known competing financial interests or personal relationships that could have appeared to influence the work reported in this paper.

## Abbreviations

ACE2: Angiotensin-converting enzyme 2
ARDS: acute respiratory distress syndrome
BMI: body mass index
CRRT: continuous renal replacement therapy
CT: computed tomography
COVID-19: Coronavirus Disease 2019
CI: confidence interval
CKD: chronic kidney disease
FiO2: fraction of inspired oxygen
IBW: ideal body weight
ICU: intensive care unit
INR: international normalized ratio
MV: mechanical ventilation
PEEP: Positive end-expiratory pressure
PIP: peak inspiratory pressure
PTT: partial thromboplastin time
RRT: renal replacement therapy
SARS-CoV-2: severe acute respiratory syndrome coronavirus 2
V–V ECMO: venous extracorporeal membrane oxygenation
